# Formative Evaluation of CLABSI Adoption and Sustainment Interventions in a Pediatric Intensive Care Unit

**DOI:** 10.1097/pq9.0000000000000719

**Published:** 2024-04-03

**Authors:** Lindsey J. Patton, Angelica Morris, Amanda Nash, Kendel Richards, Leslie Huntington, Lori Batchelor, Jenna Harris, Virginia Young, Carol J. Howe

**Affiliations:** From the * Children’s Critical Care Services and Nursing Research, Dallas, Tex.; † Harris College of Nursing, Texas Christian University, Fort Worth, Tex..

## Abstract

**Background:**

Pediatric patients require central venous catheters to maintain adequate hydration, nutritional status, and delivery of life-saving medications in the pediatric intensive care unit. Although central venous catheters provide critical medical therapies, their use increases the risk of severe infection, morbidity, and mortality. Adopting an evidence-based central line-associated bloodstream infection (CLABSI) bundle to guide nursing practice can decrease and sustain low CLABSI rates, but reliable and consistent implementation is challenging. This study aimed to conduct a mixed-methods formative evaluation to explore CLABSI bundle implementation strategies in a PICU.

**Methods:**

The team used The Consolidated Framework for Implementation Research to develop the interview guide and data analysis plan.

**Results:**

Facilitators and barriers for the CLABSI bundle occurred in four domains: inner setting, process, characteristics of individuals, and innovation characteristics in each cycle that led to recommended implementation strategy opportunities. The *champion* role was a major implementation strategy that facilitated the adoption and sustainment of the CLABSI bundle.

**Conclusions:**

Implementation Science Frameworks, such as Consolidated Framework for Implementation Research (CFIR), can be a beneficial framework to guide quality improvement efforts for evidence-based practices such as the CLABSI bundle. Using a champion role in the critical care setting may be an important implementation strategy for CLABSI bundle adoption and sustainment efforts.

## INTRODUCTION

Approximately five million central venous catheters (CVCs) are placed in hospitalized patients each year.^1,2^ Although CVCs provide critical therapies, their use increases the risk for severe morbidity and mortality.^3–6^ In 2008, The Joint Commission reported approximately 80,000 central line-associated bloodstream infections (CLABSI) in ICUs.^6^ Thirteen years later, there were still over 30,389 CLABSIs reported in over 3700 hospitals.^7^ Research has demonstrated a reduction of CLABSI by introducing standardized CLABSI prevention bundles,^8–11^ yet sustaining low CLABSI rates remains challenging. Woods-Hill and colleagues^12^ found that nurses’ CLABSI knowledge was strong, but they experienced many barriers, including lack of time, bundle workflow, lack of personnel, and parent refusal. In contrast, facilitators included lower patient acuity, patient volume, and additional help. Commonly used CLABSI prevention bundle implementation strategies in the adult literature include educational activities, reminders, audit and feedback, quality management, planning, and restructuring.^13^ Implementing evidence-based practices, such as the CLABSI prevention bundle, is often complex and requires more than one implementation strategy to effectively sustain.^14^

Most CLABSI bundle efforts have used quality improvement methodology, however, implementation science (IS) methods that identify and address barriers and facilitators may accelerate and spread the adoption and sustainment of the CLABSI bundle. The field of IS focuses on the factors that influence the uptake, implementation, and sustainment of evidence-based interventions, such as the CLABSI prevention bundle, to close the gap between what is known and what is performed in practice.^15^ Damschroder et al^16^ developed the Consolidated Framework for Implementation Research (CFIR) from a synthesis of 69 dissemination and implementation theories that define 39 constructs that may act as determinants of innovation across 5 domains: intervention characteristics, outer setting, inner setting, individual characteristics, and processes.^17^

Since 2019, the study sites’ PICU used several implementation strategies to meet their CLABSI rate goal of less than or equal to 1.3 infections per 1000 central line days. Despite many efforts, the PICU experienced ongoing challenges of nurses sustaining standardized maintenance practices. The PICU developed a CLABSI team of bedside clinical PICU nurses that acted as *champions* who conducted *educational meetings*, provided *ongoing training*, rounded on patients with CVCs to provide *audit and feedback* with a CLABSI bundle *checklist for quality monitoring,* and held monthly CLABSI *team meetings*.^18^

The purpose of this mixed methods study was to perform a formative evaluation of implementation strategies for the adoption and sustainment of the CLABSI bundle. The specific aims were to explore the perceptions of the nurses, CLABSI team, and unit leaders about the CLABSI bundle implementation strategies and to identify perceived facilitators and barriers to CLASBI bundle adoption and sustainment.

## METHODS

### Study Design

We used a mixed methods research design for formative evaluation of implementation strategies to implement and sustain CLABSI bundle practices intended to reduce CLABSI rates.

### Study Setting and Sample

The setting was a PICU spanning two floors, with 44 licensed beds and approximately 180 nurses. The PICU is within a Magnet-designated, academic pediatric hospital system located in the Southwest with a level one trauma designation. The average monthly CVC days were 440. The study sample included the nursing leaders, PICU nursing staff, and the CLABSI team who had cared for at least one patient with a CVC and nursing leaders having management responsibilities in the PICU.

### The Research Team

The co-investigators included the Director of Nursing Research and a nursing faculty. The co-investigators provided a 2-h training on the purpose of the study and qualitative interviewing techniques to a nurse leader and a clinical nurse who aided in data collection.

### Data Collection

**Fig. 1.**
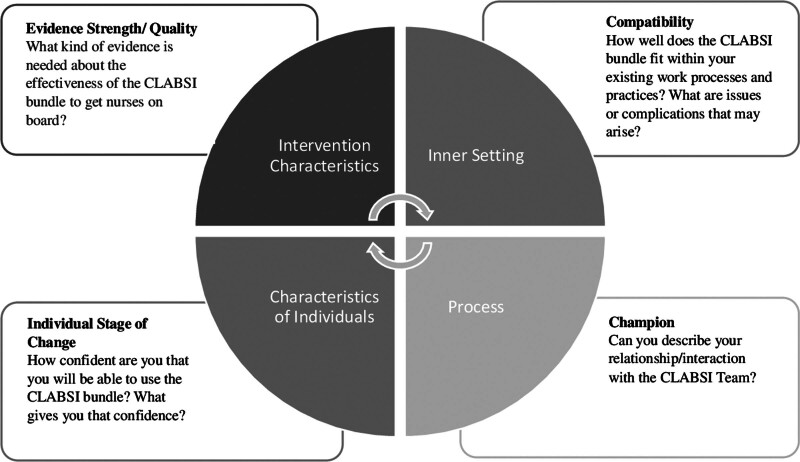
CFIR: interview guide domains. This figure demonstrates how the qualitative interview guide was developed utilizing the CFIR domains and constructs.

The co-investigators developed an interview guide, with input from the CLABSI team, with selected CFIR domains and (sub) constructs relevant to CLABSI bundle implementation (Fig. 1). Researchers divided the study into 4 cycles every 3 to 6 months to collect interview data, analyze data, and provide formative evaluation back to the CLABSI team for implementation. Study team members conducted individual interviews with PICU nurses and group interviews during scheduled CLABSI team and PICU leadership meetings; nurses participated in one interview per cycle and could participate in an interview in each study cycle. Sampling continued each cycle until thematic saturation occurred (ie, when no new themes emerged in the interviews).^19^ The research team observed CLABSI team rounds and took notes to validate themes emerging from the interviews. In each cycle, the research team collected CLABSI and CLABSI bundle compliance rates from the CLABSI team. The PICU calculated CLABSI rates as the number of CLABSI events divided by 1000 catheter days. The CLABSI team audited the CLABSI bundle on nine components and calculated a monthly compliance rate with each component. The CLABSI bundle compliance rate was calculated by averaging each of the nine audited bundle elements for an overall average compliance each month.

### Data Analysis

Interviews were audio-recorded, transcribed verbatim, and uploaded into a qualitative analysis software. A directed content analysis approach using the CFIR framework guided the qualitative analysis^20^ in three iterative steps: (1) read each transcript several times to immerse in the data; (2) establish a priori categories using the 5 CFIR domains; (3) use CFIR constructs as next level codes. We used thematic saturation, use of analytic and code memos, and member checking, a process of returning to the participants for feedback on codes to ensure trustworthiness of the qualitative data and analysis.^21^

## RESULTS

**Fig. 2.**
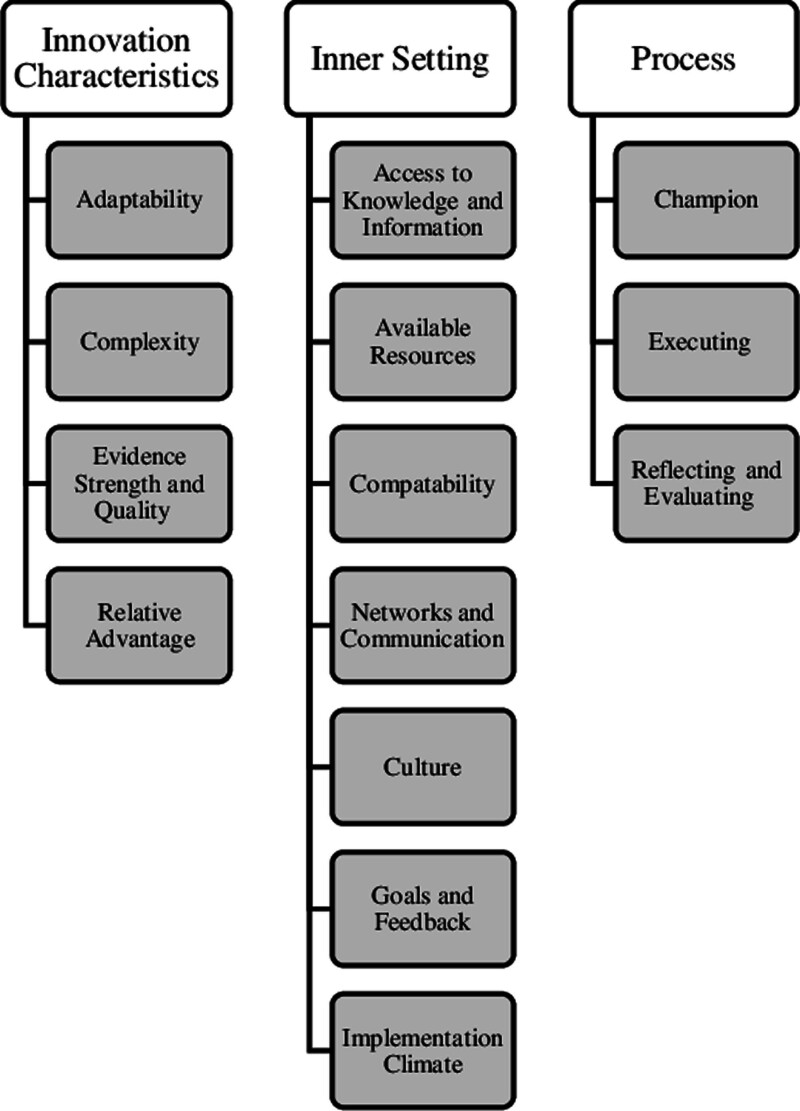
CFIR domains and constructs. This figure outlines the three CFIR domains and 14 constructs that resulted from this study.

We conducted four cycles of interviews. The sample included 62 nurses with 4 group interviews and 37 individual interviews (Table 1). We identified facilitators and barriers for the CLABSI bundle in three primary CFIR domains and 14 CFIR constructs (Fig. 2). Opportunities were fed back to the CLABSI team and leaders each cycle to evaluate for an implementation strategy (Table 2).

**Table 1.** Participant Demographics

	Individual Interviews (N = 37)	Group Interviews (N = 25)	Total (N = 62)
	*n* (%)	*n* (%)	*n* (%)
Cycle One	9 (24.3%)	Group 1: 9 (36%) Group 2: 4 (16%)	22 (35.5%)
Cycle Two	6 (16.2%)	Group 3: 5 (20%) Group 4: 7 (28%)	18 (29.0%)
Cycle Three	17 (46.0%)	0	17 (27.4%)
Cycle Four	5 (13.5%)	0	5 (8.1%)

**Table 2.** Facilitator and Barrier Opportunities Identified

Cycle	Opportunities
**One**	• Focus on individual nurses to clarify and reinforce CLABSI bundle [eg, CLABSI team members each assigned to a nurse cohort verses rounding on the patients with CVCs only (*process*)].* • Collaborate with bedside staff nurses to proactively anticipate issues with CLABSI bundle intervention for unstable patients to build an individualized plan of care (*process*). • Process map CLABSI bundle elements to clarify workflows between night and day shift (*inner setting*).
**Two**	• Continue auditing and feedback with light duty nurses for the CLABSI team (*process*).* • Highlight staff nurses’ success with the CLABSI bundle (*process*).* • Create a formal consult for nurses to access the CLABSI team (*characteristics of individuals*).*
**Three**	*•* Create guidelines for staff nurses to perform the CLABSI bundle for unstable patients (*innovation characteristics*). *•* Deconstruct unit-level CLABSI data for staff nurses into a user-friendly format and highlight nurse successes with CLABSI bundle (*process*). *•* Take advantage of opportunities on the unit to engage staff nurses and travellers on CLABSI bundle education (*process*)
**Four**	• Sustain CLABSI bundle champion as the quality nursing role (process)

* Opportunities presented to CLABSI team that could be implemented in some capacity.

## QUALITATIVE RESULTS

### Innovation Characteristics Domain

PICU nurses reported mixed perceptions of the *strength and quality of evidence* for the CLABSI bundle. Although some elements of the CLABSI bundle (eg, scrubbing the hub and keeping the dressing clean, dry, and occlusive) had strong evidence to support the practice, others lacked supportive data (eg, bathing and linen change). A CLABSI team member spoke of a demonstration that reinforced the research evidence,

*I vividly remember a presentation that showed when you scrub a hub for 5 seconds, 10 seconds, 15 seconds, and it had a black light…you could really see. Those kinds of things stick with me.*

Nurses identified that the CLABSI bundle was of low *complexity* but were unsure about the *adaptability* of the bundle (eg, not changing the linens daily) for unstable patients. Nurses reported making their own judgments on the risk/benefit of skipping bundle elements without knowing how this affected CLABSI risk. As one nurse reported,

*if they can’t tolerate a turn because they’re so unstable and on pressers, that it’s literally called a death turn...that would be a reason to not do the full linen change or to do a full CHG bath.*

### Inner Setting Domain

Participants discussed issues with the *compatibility of* existing workflows as a barrier to complete the CLABSI bundle. Although participants relayed usual responsibilities for day and night shift, some seemed unsure how to adapt the bundle depending on patients’ needs. For example, day shift nurses were typically responsible for cap and tubing changes, but night shift was responsible for changing TPN. As one nurse described,

*there’s some confusion if they are on TPN and lipids and we’ll just leave the cap and tubing for night shift.. it seems like a waste of a cap change kit so I think definitely some clarification would be good.*

Nurses also discussed the need to time CLABSI bundle elements based on patient needs rather than inflexible rules of day versus night shift responsibilities.

*You have to trust that teammates are handing off to each other about what was done and what was not done and not assuming that it was the other shift’s responsibility.*

In all cycles, participants reported that goals and feedback for completing CLABSI bundle elements and CLABSI rates were regularly communicated to staff. Once a shift, an audit was completed on a randomly chosen patient. Results of this audit (ie, passed or not) were placed on a local safety board for staff awareness. PICU leadership sent a monthly email reporting CLABSI rates. *Leadership engagement was evident throughout all the cycles.* In Cycle 1, nursing leaders paid the CLABSI team (*champion*) their hourly wage to come in additional hours and audit and round on CVCs. In later study Cycles, as CLABSI team members focused on direct patient care, leaders collaborated with the CLABSI team to identify ways to continue audits and rounding. First, leadership appointed light-duty nurses and later created a newly established role called the quality champion nurse to assume auditing and rounding for all hospital acquired conditions. One PICU leader expressed the rationale behind their support for the CLABSI efforts,

*The PICU has historically had 10 to 12 CLABSIs a year, so hopefully we’re changing that trend. We spend a little bit of money on the front side, spending the effort and less money on the backside by preventing harm to kids.*

### Process Domain

The CLABSI team (*champion*) evolved throughout the study to meet the PICU’s needs for CLABSI bundle sustainment. In Cycle 1, study participants viewed the CLABSI team as content experts who regularly rounded completed audits, and supported staff. They kept up with current literature and best practices, actively and passively disseminating information during rounds, staff meetings, education sessions, emails, and tip sheets. As the clinical nurse specialist described,

*We have more content experts within the staff and just really supporting them and being able to just share that information and knowledge that we’re gaining.*

Initially, the CLABSI team was a voluntary position in the PICU with leadership providing dedicated time for CLABSI efforts. They set the CLABSI bundle implementation tone by rounding and auditing during extra four-hour shifts. Rounding increased the CLABSI teams’ presence and ability to provide real-time feedback. For example, if the tubing was not changed or was due to be changed, the CLABSI team member assisted the staff nurse in real-time and ensured that it was properly documented. The team was responsive to questions that arose from staff during rounds. When nurses lacked knowledge on how to document the CLABSI bundle in the electronic health record, the CLABSI team partnered with the bedside staff to connect the CLABSI bundle with workflow. However, a major barrier to the CLABSI team was the inability to reach every nurse since their presence was limited to voluntary shifts.

During Cycle 2, the needs of the PICU changed. With high census, high patient acuity, and staff shortages, the PICU began providing incentive pay for staff to work direct patient care shifts, resulting in a drift of the CLABSI team’s efforts to round and audit. Nurse participants reported a lack of the CLABSI team’s presence with auditing and feedback. Despite a light duty staff nurse who was trained to round and audit, there was a decrease in tracking compliance to the CLABSI bundle during Cycle 2.

In Cycle 3, the PICU leadership recognized the importance of the presence of the CLABSI team on the unit and partnered with the CLABSI team to design a new quality champion nurse role. One CLABSI team member expressed,

*the CLABSI team couldn’t get to the rounding because the patient population and the patient acuity was so high, rounding started to drop off... that’s when we could see the difference between actually rounding and not rounding. We started seeing an uptake in our infection rates again. And so the quality nurse champions started doing what CLABSI team had been doing.*

During Cycle Four, the PICU had fully adapted the CLABSI team role into the quality champion nurse. Each shift, a staff nurse, removed from patient assignments, assumed the quality champion role to round with nurses to address quality concerns. Due to the success of the CLABSI team, the quality champion nurse role-modeled the same behaviors but had a broader scope over more hospital-acquired conditions. The quality champion nurses held designated phones and were available every shift for staff nurses. The CLABSI team continued to facilitate the CLABSI bundle by meeting regularly with leadership to evaluate audit data and to troubleshoot unit barriers. A CLABSI team member stated:

*Having the quality nurse is a position that isn’t only for CLABSI, but also covers the different quality hospital-acquired conditions (HAC) and they’re here for the 12-hour shift, so they’re available to do those line audits. They’re available to help with dressing changes. And so if somebody is having a problem with or needs assistance with a dressing change, they can help with that. They’re not in clinical staffing numbers. As opposed to when our CLABSI crew members, were coming in on their off days.*

### Quantitative Data

**Fig. 3.**
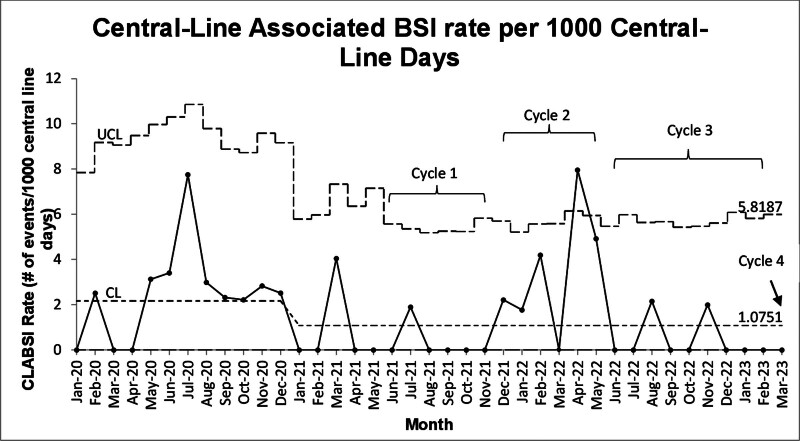
PICU central-line-associated blood stream infection rates U-chart. The solid line represents (in black) CLABSI rates. Rates are calculated as number of CLABSI events divided by 1000 catheter days. The central dashed line represents the mean CLABSI rate. The upper dashed line is the upper control limit (UCL), and the lower dashed line is the lower control limit (LCL), set at zero. Each study cycle is also displayed.

**Fig. 4.**
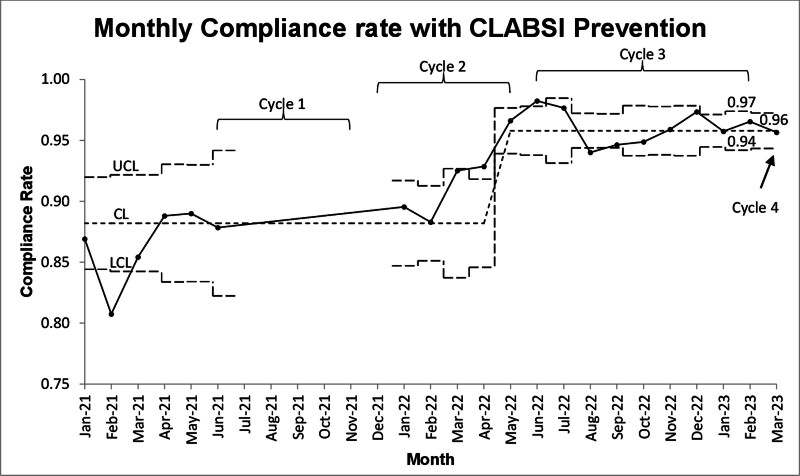
PICU central-line-associated blood stream infection bundle compliance rates U-chart. This figure displays overall CLABSI bundle compliance each month (solid black line). The central dashed line represents the mean CLABSI bundle compliance rate. The upper dashed line is the upper control limit (UCL), and the lower dashed line is the lower control limit (LCL). Each study cycle is also displayed. There are missing data from July ’21 to Jan ’22 due to insufficient staff to collect the data.

Throughout the study, the monthly CLABSI rate (Fig. 3) and bundle compliance (Fig. 4) were monitored by study personnel. The CLABSI rate has one centerline shift in January 2021 and the mean rate remained at 1.07 throughout the rest of the study. There were no CLABSI rate changes during any of the four cycles (Fig. 3). Bundle compliance increased significantly to above 95% in May 2022 (centerline shift). There was missing data from July 2021 through December 2021 as the CLABSI team experienced significant strain.

### Discussion

Sustained efforts for CLABSI reduction remain challenging for many PICUs. Pediatric collaboratives have found significant impacts on CLABSI reduction and sustainability over time with maintenance bundle elements.^22^ However, data regarding how PICUs hardwire and sustain high bundle compliance and implementation strategies with nursing staff overtime are limited. The purpose of this study was to explore perceptions of the CLABSI bundle to identify and address facilitators and barriers to CLABSI bundle adoption and sustainment. Previous studies revealed a statistically significant reduction in CLABSIs with adherence to CLABSI maintenance bundles.^22,23^ In contrast, this study found that CLABSI rates remained low even during times when compliance auditing was not possible because of staffing constraints. We cautiously interpret these findings to suggest that the CLABSI champion, viewed as a clinical expert, promoted awareness of the CLABSI bundle that in turn maintains low CLABSI rates. Similarly, previous studies found lower CLABSI rates with the presence of champions who frequently rounded, used audit and feedback, and acted as a resource.^24–26^

In the real-world setting of the PICU, there were barriers to the fidelity of implementing the champion role. The initial intent in this study was for the CLABSI team to select and implement one or two opportunities identified by the evaluation team each study cycle. Themes and opportunities emerged from each study cycle drawing insight into the PICU’s efforts to reduce CLABSIs. However, many barriers arose due to the nurse staffing shortages, high-patient acuities, and unit morale. Although this was disappointing, the inability to act on the feedback confirmed reports from a recent systematic review and meta-synthesis, finding that when staff had less capacity because of limited staffing and time (*resources*), they were less able to respond to feedback.^24^

Implementation and adherence to a CLABSI bundle required leadership support and influence to modify and adapt the staff and needs to the challenges that the PICU may face. The unit leadership responded to challenges by collaborating with the CLABSI team to create an expanded quality champion role, confirming evidence that implementation efforts continued when leadership advocated for resources.^26,27^ As the CLABSI team experienced barriers, the PICU leadership necessarily became involved to assist with removing barriers and supporting efforts. Leadership support was an essential facilitator for the adoption and long-term sustainability of evidence-based practices^27^ such as the CLABSI bundle.

### Limitations

This study has several limitations. First, the research team conducted interviews with a convenience sample of nurses available on data collection days, which may not represent the perceptions of all nurses in the PICU. The team, however, conducted interviews with a purposive sample of CLABSI team members and unit leaders who were most informed about the CLABSI bundle and unit operations. Second, interview data relied on nurses’ self-report, which was potentially subject to selective memory bias and social desirability bias; to minimize these biases, the research team were not part of the PICU staff and interview questions were located in present time. Third, the data collection and analysis may have been subject to researcher bias. On the other hand, the expertise of the principal investigator with CLABSI prevention and the CLABSI bundle may have allowed a deeper understanding and interpretation of interview data. Additional limitations included the inability of the CLABSI champion to consistently collect compliance data on the CLABSI bundle and the lack of a historical timesheet to calculate CLABSI champion hours (dose) dedicated to implementation efforts. Because the study was performed within a single PICU in a single institution, the reader is encouraged to consider the transferability of findings to their clinical setting.

### Future Research

Opportunities to further study implementation strategies were easily identified using an IS framework; however additional work is needed to understand the effectiveness of specific implementation strategies for CLABSI bundle sustainability. Although many studies focus on the champion role, future research should focus on the dose, fidelity, and development of the champion role for implementation, adoption, and sustainment of evidence-based practices.

## CONCLUSIONS

The CLABSI bundle is a familiar and easily understood PICU nursing practice, but sustainment efforts require active interventions targeted at the process and inner setting domains. A champion role may be an important implementation strategy that PICUs can use to influence the adoption and sustainment of evidence-based practices. Leadership involvement and engagement are important antecedents for the champion to overcome barriers to implementation. This study’s findings underscore the importance of fostering a culture of accountability and empowerment of bedside nurses to act as champions who can inspire their colleagues to consistently follow evidence-based practices that ultimately result in improved patient safety and outcomes.

## ACKNOWLEDGMENTS

The authors would like to acknowledge Marshall Stephenson, BSN, RN, CCRN-K, Vice President, Critical Care Services at Children’s Medical Center Dallas for his support of the study and the Nursing Research and Evidence-based Practice Program at Children’s Health System of Texas for their support.
